# Diagnostic value of echocardiography combined with serum C-reactive protein level in chronic heart failure

**DOI:** 10.1186/s13019-023-02176-7

**Published:** 2023-03-25

**Authors:** Yongxia Zhang

**Affiliations:** Cardiovascular Medicine Department, The Third Affiliated Hospital of Guangzhou Medical University, No.63 Duobao Road, Liwan District, Guangzhou, 510150 Guangdong Province China

**Keywords:** Echocardiography, C-reactive protein, Chronic heart failure, Combined diagnosis, Receiver operating characteristic curve, Serum, Logistic regression

## Abstract

**Background:**

Chronic heart failure (CHF) is regarded as common clinical heart disease. This study aims to investigate the clinical diagnostic value of echocardiography (Echo) and serum C-reactive protein (CRP) levels in patients with CHF.

**Methods:**

A total of 75 patients with CHF (42 males, 33 females, age 62.72 ± 1.06 years) were enrolled as study subjects, with 70 non-CHF subjects (38 males, 32 females, age 62.44 ± 1.28 years) as controls. The left ventricular ejection fraction (LVEF), fraction shortening rate of the left ventricle (FS), and early to late diastolic filling (E/A) were determined by Echo, followed by an examination of the expression of serum CRP by ELISA. In addition, the Pearson method was used to analyze the correlation between echocardiographic quantitative parameters (EQPs) (LVEF, FS, and E/A) and serum CRP levels. Receiver operating characteristic (ROC) curve was adopted to evaluate the diagnostic efficacy of EQPs and serum CRP levels for CHF. The independent risk factors for CHF patients were measured by logistics regression analysis.

**Results:**

The serum CRP level of CHF patients was elevated, the values of LVEF and FS decreased, and the E/A values increased. ROC curve revealed that the EQPs (LVEF, FS, and E/A) combined with serum CRP had high diagnostic values for CHF patients. Logistic regression analysis showed that the EQPs (LVEF, FS, and E/A) and serum CRP levels were independent risk factors for CHF patients.

**Conclusion:**

Echo combined with serum CRP level has high clinical diagnostic values for CHF patients.

## Background

Chronic heart failure (CHF) is a clinical syndrome of ventricular filling dysfunction and ejection dysfunction due to cardiac structural damage and functional disturbances caused by various factors, including ischemic heart disease, hypertension, and cardiomyopathy [1, 2]. Although substantial improvements have been made in most heart diseases, heart failure (HF) remains a major health problem with an increasing incidence and prevalence over the past few decades [3]. In particular, the ongoing improvement of other cardiovascular diseases (such as myocardial infarction), the aging of the population, and the prevalence of comorbidities have all contributed to the increased incidence of CHF [4]. Yet, patients with HF commonly present signs and symptoms that are nonspecific and widely differentiated, making diagnosis by clinical presentation alone challenging [5]. Accordingly, early and accurate diagnosis and treatment are essential to lower the morbidity and mortality of CHF.

Echocardiography (Echo) is a diagnostic method for assessing the systolic and diastolic function of the heart to diagnose and manage HF [6]. In parallel, Echo can provide extremely important data through the evaluation of cardiac function to understand the underlying mechanism of hemodynamic instability of patients, even in the case of cardiopulmonary arrest, and can swiftly establish an appropriate treatment plan for patients [7, 8]. With organized methods of two-dimensional Echo and Doppler Echo, clinicians can determine left ventricular systolic and diastolic function and estimate cardiac output, pulmonary artery, and ventricular filling pressure [9, 10]. Moreover, real-time three-dimensional Echo provides unprecedented volume data to quantify the left ventricular state [11]. Expert assessment of symptoms and signs coupled with objective investigations, including Echo, is the gold standard for diagnosing left ventricular systolic dysfunction (LVSD) and progressively suspected HF with a preserved ejection fraction [12]. In a word, Echo plays an important role in the diagnosis of CHF [13]. Nevertheless, manual interpretation of Echo can be time-consuming and prone to human error [14].

C-reactive protein (CRP) has been established as a classic marker of systemic inflammation [15]. It is produced principally by liver cells, but also by cardiovascular tissues in response to infection, cell invasion, or tissue injury [16]. Cumulative evidence confirms the presence of local and systemic inflammation in HF patients and reports that elevated serum CRP levels are significantly associated with cardiovascular events and mortality [17–19]. Additionally, the ability of CRP to predict vascular risk means that it may be used as a biomarker to identify individuals, particularly benefiting from risk reduction treatments [20]. However, to date, clinical studies on the diagnosis of CHF by Echo combined with serum CRP have not been reported, and further exploration is needed. The purpose of this study was to investigate the clinical significance of Echo combined with serum CRP level in the diagnosis of CHF.

## Materials and methods

### Ethics statement

This study was ratified by the Ethics Committee of The Third Affiliated Hospital of Guangzhou Medical University, and each subject signed the informed consent. All procedures were strictly implemented according to the Declaration of Helsinki.

### Study subjects

A total of 75 CHF patients (42 males and 33 females, aged 62.72 ± 1.06 years) diagnosed and treated in The Third Affiliated Hospital of Guangzhou Medical University from June 2018 to June 2020, were assigned to the study group (CHF group). During the same period, 70 non-CHF subjects (38 males and 32 females, age 62.44 ± 1.28 years) matching the age and sex of the study group were enrolled as the control group.

Inclusion criteria [21]: (1) All patients were diagnosed with HF for more than six months, and the diagnosis of CHF was based on the European Society of Cardiology Guidelines for the Diagnosis and Treatment of Acute and Chronic Heart Failure 2012 [22]; (2) Patients with complete clinical data; (3) Patients who received HF treatment for 1 month or more after the beginning of the study; (4) Patients over 60 years of age. Exclusion criteria: (1) with acute coronary syndrome before admission; (2) with congenital heart disease, pulmonary heart disease, severe heart valvular disease; (3) with severe liver and kidney failure; (4) with incomplete examination data; (5) with malignant tumors.

Cardiac function was graded according to the New York Heart Association (NYHA) grading system revised in 1928 and 1994 [23]. Class I: the patient has heart disease but is not restricted in daily activities; normal activity does not cause fatigue, palpitations, dyspnea, and angina. Class II: the patient has heart disease, with a slight limitation of daily activities after normal work and no symptoms after a short rest, but daily activities may cause mild dyspnea, palpitations, angina pectoris, fatigue, and other symptoms. Class III: patients with heart disease have significantly limited daily activity, even if few daily activities can cause a series of symptoms. Class IV: patients with heart disease are incapable to engage in any mild physical activity and may develop symptoms of HF while at rest; mild physical activity may worsen the symptoms of HF.

### Data collection

Age, sex, body mass index (BMI), smoking history, drinking history, total cholesterol (TC), triglyceride (TG), low-density lipoprotein cholesterol (LDL-C), high-density lipoprotein cholesterol (HDL-C), uric acid (UA), left ventricle ejection fraction (LVEF), complications (hypertension, diabetes) and other clinical baseline data of the enrolled subjects were recorded, as well as NYHA cardiac function classification of the patients. The 3 mL of elbow venous blood was extracted from the enrolled CHF patients on an empty stomach on the morning of the second day after admission. Similarly, blood samples were collected from control subjects and centrifuged at 2800 × g for 10 min. The supernatant was collected and stored in a refrigerator at − 80 °C for centralized detection.

### Enzyme-linked immunosorbent assay (ELISA)

The serum CRP level of the subjects was examined using a double antibody sandwich ELISA kit (ab260058, Abcam, Cambridge, UK), and the specific operation was carried out under the kit instructions. In short, CRP was measured by ELISA using a 100-fold diluted serum sample at a final volume of 50 μL. Samples were added to a 96-well micro-titration plate precoated with CRP-specific monoclonal antibody and incubated for 2 h. After four times of washing, the serum was incubated with an anti-CRP antibody conjugated with horseradish peroxidase for 2 h for detection. The titration plate was washed four times and incubated with the substrate solution for 30 min. Eventually, 50 μL of stop solution was supplemented to each well, and the color altered from blue to yellow. Optical density (OD) values of each well were determined at 450 nm using a microplate reader (Tecan, Mannedorf, Switzerland). The results were linearized by plotting the logarithm (log) of the CRP concentration versus the log of the OD value, and the best fitting line was measured by regression analysis. Since the samples were diluted previously, the concentrations read from the standard curve were corrected by the dilution factor.

### Examination of patient indicators by Echo

Cardiac function was evaluated in subjects in both groups, and Philips Affiniti 70W (IL, USA) Doppler echocardiography was used for cardiac ultrasound examination, with the frequency parameters of the instrument probe set to 2 ~ 4 MHz. Detailed operation methods were as follows: the patient was in a left lateral decubitus position; after the probe was smeared with the coupling agent, the probe was placed in the chest apex of the patient and the inspection screen was observed; according to the cardiac structure of the patient, the inspectors need to move the position of the inspection probe back and forth, and switch the direction of the probe to observe the cardiac valve structure of the patient in detail and obtain clear images. Inspectors also need to switch the function of the ultrasound examination instrument to test the left ventricular ejection fraction (LVEF), fraction shortening rate of the left ventricle (FS), and early to late diastolic filling (E/A), to obtain more accurate test results.

### Statistical analysis

SPSS21.0 statistical software (IBM Corp. Armonk, NY, USA), GraphPad Prism 6.0 software (GraphPad Software Inc., CA, USA), and MedCalc 19.0 Software (MedCalc Software Ltd, Ostend, Belgium) were used for statistical analysis and plotting of data. Shapiro–Wilk test was used for the normal distribution test, measurement data in normal distribution was expressed as mean ± standard deviation (SD), and data comparison between groups was performed by the *t* test; counting data were represented as the number of cases, and the chi-square test was used for data comparison between groups. ROC curve was used to evaluate the diagnostic efficiency of the parameters, and the optimal ROC curve threshold was found by using the Youden index. Logistic model was used for multivariate regression analysis. *P* was obtained from the bilateral tests. A value of *P* < 0.05 meant statistical significance.

## Results

### Clinical baseline characteristics of the subjects

A total of 75 CHF patients and 70 matched non-CHF subjects were recruited. Their basic clinical information is shown in Table 1. There were no significant differences in age, sex, BMI, drinking history, TG, HDL-C, hypertension, and diabetes between the two groups, but significant differences in smoking history, TC, LDL-C, and UA (all *P* > 0.05). Meanwhile, compared with the control group, LVEF (%) and FS (%) values in the CHF group were substantially reduced, while E/A values were enhanced (all *P* < 0.001).

**Table 1:** Comparative analysis of clinical baseline data between CHF patients and non-CHF subjects

Feature	Control (N = 70)	CHF (N = 75)	*P* value
Age (year)	62.44 ± 1.28	62.72 ± 1.06	0.157
Gender (male/female)	38/32	42/33	0.836
BMI (kg/m^2^)	23.58 ± 0.34	23.62 ± 0.37	0.501
Smoking history (never/ever)	57/13	48/27	0.005
Drinking history (never/ever)	48/22	40/35	0.418
TC (nM)	4.75 ± 0.22	4.67 ± 0.18	0.019
TG (nM)	1.54 ± 0.52	1.47 ± 0.46	0.394
LDL-C (nM)	2.86 ± 0.27	2.96 ± 0.32	0.044
HDL-C (nM)	1.25 ± 0.24	1.24 ± 0.23	0.806
UA (µM)	349.28 ± 15.32	354.74 ± 16.20	0.039
*Complication (no/yes)*			
Hypertension	45/25	53/22	0.412
Diabetes	47/23	57/18	0.236
*EQP*			
LVEF (%)	48.74 ± 7.62	39.48 ± 5.64	< 0.001
FS (%)	28.16 ± 4.09	22.37 ± 3.06	< 0.001
E/A	1.17 ± 0.21	1.42 ± 0.22	< 0.001
*NYHA stage*			
I	–	14	–
II	–	24	–
III	–	25	–
IV	–	12	–

Measurement data were expressed as mean ± SD, and *t* test was used for data comparison between two groups; counting data were represented as the number of cases, and the chi-square test was used for data comparison between groups

*BMI* body mass index, *TC* total cholesterol, *TG* triglyceride, *LDL-C* low-density lipoprotein cholesterol, *HDL-C* high-density lipoprotein cholesterol, *UA* uric acid, *LVEF* left ventricle ejection fraction, *FS* fraction shortening rate of left ventricle, *E/A* early to late diastolic filling, *NYHA* New York Heart Association, *CHF* chronic heart failure, *EQP* quantitative parameters of Echo

### The CRP level was highly expressed in the serum of CHF patients

**Fig. 1:**
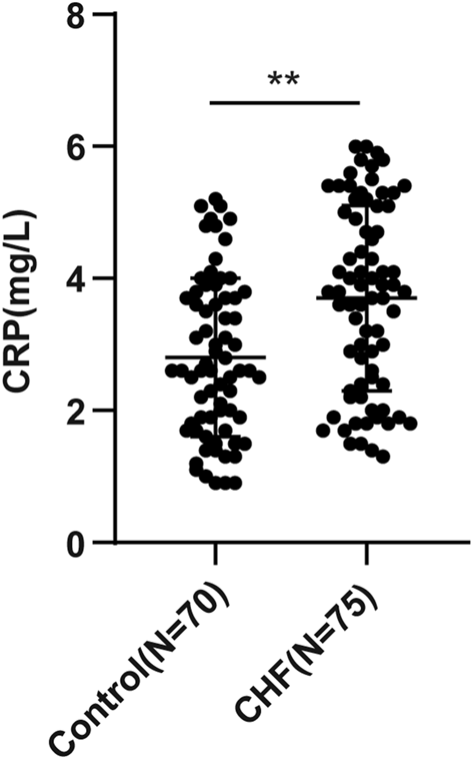
CRP was highly expressed in the serum of CHF patients. CRP expression in the serum of CHF patients and non-CHF subjects was determined by ELISA. Values were expressed as mean ± SD, and *t* test was used for data comparison between two groups. **P < 0.01

Subsequently, we identified CRP expression in the serum of CHF patients and non-CHF subjects by ELISA, which manifested that the serum CRP level of CHF patients (3.7 ± 1.4 mg/L) was higher than that of non-CHF subjects (2.8 ± 1.2 mg/L) (Fig. 1, *P* < 0.01).

### Correlation between EQPs and serum CRP levels in CHF patients

**Fig. 2:**
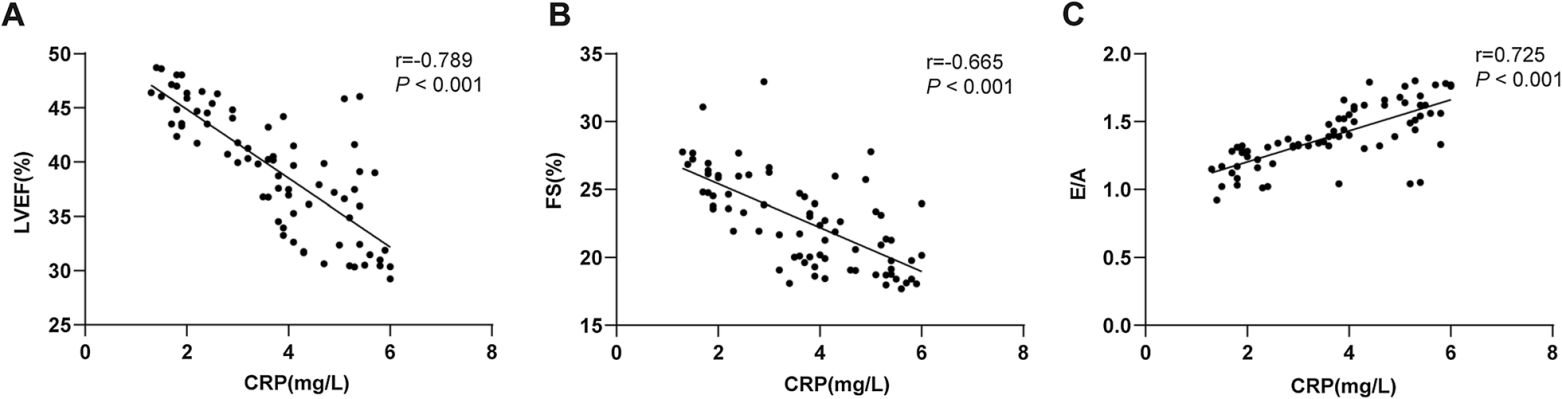
Correlation analysis of EQPs (LVEF, FS and E/A) and serum CRP level in CHF patients. Pearson method was used to analyze the correlation between LVEF and serum CRP level (**A**), FS and serum CRP level (**B**), and E/A and serum CRP level (**C**) in CHF patients

To further figure out whether EQPs are correlated with serum CRP levels in CHF patients, the Pearson method was used to conduct a correlation analysis between EQPs (LVEF, FS, and E/A) and serum CRP levels. The results revealed that serum CRP level in CHF patients was negatively correlated with LVEF and FS (r =  − 0.789, *P* < 0.001; r =  − 0.665, *P* < 0.001) (Fig. 2A/B), whereas positively correlated with E/A (r = 0.725, *P* < 0.001) (Fig. 2C).

### Diagnostic value of EQPs combined with serum CRP level in CHF

**Fig. 3:**
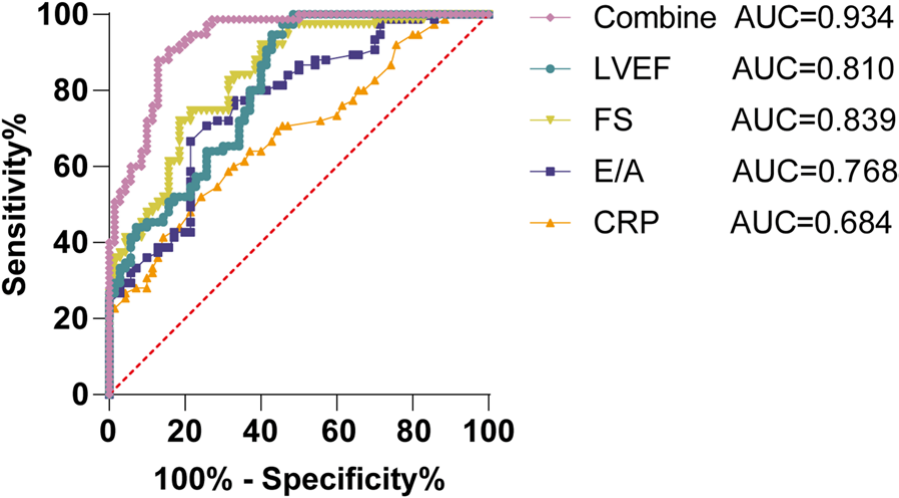
Diagnostic efficacy of EQPs combined with serum CRP on CHF. ROC curve was used to analyze the diagnostic efficacy of the EQPs (LVEF, FS and E/A), serum CRP levels, and their combination for CHF patients

To further clarify the clinical diagnostic significance of EQPs and serum CRP levels in patients with CHF, we analyzed the diagnostic efficacy of LVEF, FS, E/A, CRP level, and EQPs combined with serum CRP levels on CHF by ROC curve. The results uncovered that LVEF, FS, E/A, and CRP levels all had certain diagnostic efficacy for CHF (Table 2). MedCalc analysis illustrated that the area under the curve (AUC) of EQPs combined with serum CRP level in identifying CHF was significantly higher than that of EQPs (LVEF, FS, and E/A) or serum CRP alone (Fig. 3, all *P* < 0.05). These results indicate that EQPs combined with serum CRP levels had high diagnostic efficacy for CHF.

**Table 2:** Diagnostic efficacy of LVEF, FS, E/A, CRP and their combination for CHF

Item	LVEF	FS	E/A	CRP	Combine
AUC	0.81	0.839	0.768	0.684	0.934
95% CI	0.741–0.879	0.776–0.902	0.692–0.844	0.598–0.769	0.895–0.972
Sensitivity	94.67%	72.00%	66.67%	52.00%	88.00%
95% CI	87.07–97.91	60.96–80.90	55.42–76.29	40.87–62.93	78.74–93.56
Specificity	57.14	81.43%	78.57%	75.71%	87.14%
95% CI	45.48–68.06	70.77–88.81	67.61–86.56	64.50–84.25	77.34–93.09

### EQPs and serum CRP levels were independently correlated with the occurrence of CHF

To further investigate whether EPQs and serum CRP were independently correlated with the CHF occurrence, we conducted a logistic multifactor regression analysis with disease occurrence as the dependent variable and smoking history, TC, LDL-C, UA, EQPs (LVEF, FS, and E/A) in Table 1 (*P* < 0.1) and serum CRP level as independent variables. Logistic multifactor regression showed that after adjusting smoking history, TC, LDL-C, and UA, the EQPs (LVEF, FS, and E/A) and serum CRP level were independent correlation factors for CHF and closely correlated with CHF (Table 3).

**Table 3:** Logistic multifactor regression analysis of disease-related indicators in CHF patients

Item	*P* value	OR value	95% CI
LVEF	0.000	0.826	0.747–0.914
FS	0.000	0.706	0.594–0.840
E/A	0.027	18.321	1.381–242.977
CRP	0.041	0.545	0.304–0.976
TC	0.067	0.086	0.006–1.185
LDL-C	0.635	1.540	0.259–9.161
UA	0.355	1.015	0.983–1.048
Smoking history	0.148	2.378	0.736–7.685

## Discussion

CHF is an end-stage of multiple cardiac diseases, and its diagnosis and prognosis remain a challenge [24]. With efforts to improve the treatment and prevention of CHF, sudden death rates in CHF patients have declined substantially over the past decades [25]. Nonetheless, the overall mortality rate of these patients remains high [26]. Echo is the gold standard tool for the evaluation of patients with HF [27]. CRP concentration reflects inflammation and is currently the preferred inflammatory biomarker for cardiovascular risk stratification [28]. This study provides evidence that Echo combined with serum CRP level has high clinical diagnostic values for patients with CHF.

Patients with CHF (N = 75) and matched non-CHF subjects (N = 70) were enrolled in the study. CHF is a complex syndrome, and myocardial injury causes abnormal activation of the neuroendocrine regulatory system, resulting in sodium retention, circulatory congestion, and cardiac and vascular remodeling [29, 30]. Hence, the correct response to CHF should include hemodynamic measurement and overall cardiac function assessment [13]. Echo is a simple, reliable, low-cost, non-invasive diagnostic technique, which has been widely used in the diagnosis and assessment of CHF disease and prognosis, with the evaluation of left ventricular systolic function and diastolic function by LVEF/FS and E/A, respectively [31, 32]. In our present study, the LVEF (%) and FS (%) values of CHF patients declined, whereas E/A values were elevated, suggesting that patients with CHF had systolic and diastolic dysfunction, which was consistent with a previous study [33]. These findings and evidence highlighted the clinical diagnostic values of EQPs for CHF.

HF is characterized by systemic inflammation that worsens as the disease progresses [15]. The preferred inflammatory biomarker in cardiovascular disease is CRP [28, 34]. Numerous experimental data have demonstrated the role of inflammation in the left ventricular dysfunction and remodeling, and the occurrence and outcome of CHF [15, 35]. CRP levels were elevated in HF patients and increased with clinical decompensation, predicting a worse prognosis [19, 36]. Elevated CRP levels reflect inflammation and immune disorders in HF patients [36] and predicts deterioration of functional ability in patients with ischemic heart disease and systolic HF [37, 38]. In light of the preceding literature, our results showed a high expression of CRP in the serum of CHF patients. Previously, studies have manifested that elevated CRP can predict LVSD and left ventricular remodeling (LVR) in patients with acute segmental elevation myocardial infarction, and suggest that LVSD patients have an increased risk of HF after infarction [35, 39, 40]. Therefore, we speculated that CRP levels may have a certain correlation with EQPs (LVEF, FS, and E/A). As expected, the serum CRP level in CHF patients was negatively correlated with LVEF and FS and positively correlated with E/A, which was consistent with the reported correlation in the decompensated stage of HF in dogs [41].

All imaging techniques can provide an ejection fraction, but the versatility of Echo makes it unique in providing volumes, diastolic function, right ventricular function, hemodynamics, and valvular regurgitation [42]. Elevated serum CRP levels are significantly associated with cardiovascular events and mortality [17]. Afterwards, we analyzed the diagnostic efficacy of LVEF, FS, E/A, CRP, and their combination for CHF by ROC curves. Intriguingly, LVEF, FS, E/A, and CRP all have certain diagnostic efficacy for CHF. Notably, the diagnostic efficacy of EQPs combined with CRP for CHF was visibly better than that of EQPs or serum CRP alone. It has been reported that EQPs (LVEF, FS, and E/A) in CHF patients are correlated with vascular endothelial function, and the combination of the two can effectively forecast the risk of major adverse cardiovascular events soon [33]. To our knowledge, this is the first clinical trial to show that EQPs combined with serum CRP levels have high diagnostic efficacy for CHF.

Serum CRP level is an independent factor for HF after acute myocardial infarction [43]. Meanwhile, CRP levels have been demonstrated to predict adverse long-term clinical outcomes and cardiopulmonary health decline in symptomatic patients with chronic ischemic HF, independent of other predictors such as B-type Natriuretic Peptide [15, 44]. In addition, diminished LVEF and FS and expanded E/A are risk factors for major adverse cardiovascular events [33]. Likewise, LVEF 55% and CRP 10 mg/L are independent risk factors for pulmonary infection in patients with HF [45]. Consequently, we further considered the independent correlation between EQPs (LVEF, FS, and E/A) and serum CRP and the occurrence of CHF. In line with the above report, our experiments discovered that EQPs (LVEF, FS, and E/A) and serum CRP level were independent correlated factors for CHF.

In conclusion, as a prospective study, this paper clarified the role of EQPs (LVEF, FS, and E/A) combined with serum CRP level in the diagnosis of CHF, providing a new entry point for clinical diagnosis and classification of CHF. However, the time span of sample collection was small and the patients were older, which may affect the determination of CRP levels. In addition, few cases and EQPs were included in this study. In the future, we need to carry out multi-center prospective studies to expand the sample size and match the control, to increase the credibility of the results. Moreover, EQPs and CRP levels can be measured in the early or middle stage of CHF to study its diagnostic and prognostic values.

## Data Availability

All the data generated or analyzed during this study are included in this published article.
